# Correlation between 25-hydroxyvitamin D and severe headache or migraine: evidence from NHANES database

**DOI:** 10.29219/fnr.v68.10338

**Published:** 2024-12-09

**Authors:** Xiaolei Zhang, Jiangwen Wu, Ting Wu, Liwen Guo, Ruiping Zhang, Xin Jin

**Affiliations:** Pain Department, Xiangyang No. 1 People’s Hospital, Xiangyang City, China

**Keywords:** 25-Hydroxyvitamin D, severe headache or migraine, association, NHANES

## Abstract

**Objective:**

This study was formulated with the objective of elucidating the correlation between 25-hydroxyvitamin D (25(OH)D) and the occurrence of severe headache or migraine, employing a cross-sectional analytical approach.

**Methods:**

A cross-sectional survey was conducted over two cycles involving 7,661 participants, utilizing data from the National Health and Nutrition Examination Survey (NHANES) conducted between 2001 and 2004. A weighted logistic regression method was employed to construct a relationship model between the two variables. Subgroup analysis, adjusting for confounding factors, was performed through stratified analysis to explore the association between 25(OH)D and severe headaches or migraines. Finally, a restricted cubic spline regression (RCS) was utilized to investigate the non-linear relationship between the variables.

**Results:**

A total of 7,661 participants were included in this study, with an overall prevalence of severe headaches or migraines of 1,576/7,661 (22.3%). The results from all models consistently indicated a significant negative correlation between serum 25(OH)D levels and the risk of severe headaches or migraines (*P* < 0.05). Stratified analysis revealed that in the female population (odds ratios [OR]: 0.995, 95% CI: 0.991–0.998, *P* = 0.001), never smokers (OR: 0.991, 95% CI: 0.985–0.997, *P* = 0.003), and non-drinkers (OR: 0.993, 95% CI: 0.987–0.999, *P* = 0.022), the risk of severe headaches or migraines decreased with increasing serum 25(OH)D concentrations. RCS results demonstrated a linear relationship between serum 25(OH)D levels and the risk of severe headaches or migraines.

**Conclusion:**

We discovered a negative correlation between serum 25(OH)D levels and the prevalence of severe headaches or migraines, particularly in females, non-smokers, and non-hypertensive individuals. Further clinical research is necessary to confirm these findings, establish causality, and explore potential preventive and therapeutic mechanisms for migraines.

## Popular scientific summary

- This study investigates the link between 25-hydroxyvitamin D (25(OH)D) levels and the risk of severe headaches or migraines, using data from the NHANES (2001-2004).
- Results show that lower 25(OH)D levels are associated with a higher risk of severe headaches, particularly in females, non-smokers, and non-hypertensive individuals.
- Maintaining adequate vitamin D levels may help reduce headache risk, highlighting the importance of a healthy diet and lifestyle.
- Further research is needed to explore the benefits of vitamin D supplementation in headache management.

## Brief scientific summary

This cross-sectional study utilized data from the 2001–2004 NHANES to investigate the relationship between serum 25-hydroxyvitamin D levels and the risk of severe headaches or migraines, involving 7,661 participants. This study found a significant inverse correlation between serum 25-hydroxyvitamin D levels and the risk of severe headaches or migraines, particularly more pronounced in women, non-smokers, and non-drinkers. A linear relationship between the two was confirmed through restricted cubic spline (RCS) regression. Higher serum 25-hydroxyvitamin D levels may reduce the risk of severe headaches or migraines, warranting further clinical research to explore potential causal mechanisms and preventive strategies.

Headache is one of the major neurological disorders, with a prevalence of nearly 50% in the general population, prevalent in both adults and children, and the incidence increases with age (1). Primary headaches account for nearly 98% of all headaches, including tension-type headache (TTH), migraine, and cluster headache (2). Secondary headaches are uncommon, but early diagnosis is crucial for life-saving interventions (3). In primary headaches, migraine is a common and complex neurovascular disorder often accompanied by severe headache, nausea, vomiting, photophobia, and phonophobia (4), with pain ranging from moderate to severe (5). The term ‘severe headache’ is generally used to describe various types of intense headaches, including but not limited to migraines, and broadly refers to any strong headache sensation, which may involve TTHs, cluster headaches, or other forms of headaches. The American Migraine Prevalence and Prevention Study indicated that most individuals reporting ‘severe headache’ met the diagnostic criteria for migraine or probable migraine (6).

Migraine, as the most common primary headache disorder, is a complex, multifaceted, debilitating neurovascular disease. It is prevalent worldwide, affecting over 1 billion people globally (7, 8). Approximately 80% of migraine sufferers experience postdrome symptoms following the headache phase, which may include fatigue, impaired concentration, photophobia, and bodily pain (9). As one of the recognized over 200 types of headache disorders (10), an investigation showed that 15.3% of Americans report suffering from migraines and severe headaches, with 9.7% in males and 20.7% in females (11). This ailment frequently occurs in the young adult population aged between 18 and 44, and its widespread prevalence and detrimental physiological, psychological, and cognitive consequences impose a substantial burden on affected individuals, families, and society at large (11, 12). Therefore, it is essential to study effective preventive measures or modifiable risk factors for severe headaches or migraines.

Lately, the correlation between neurologic disorders and vitamin D has been gaining increasing attention in the scientific community (13). Vitamin D µg is poised to exert diverse impacts upon the nervous system, with its insufficiency potentially emerging as a conceivable predisposing element in the pathogenesis of myriad neurological disorders. The pivotal storage form of Vit D, 25-hydroxyvitamin D (25(OH)D), assumes a crucial role in the modulation of phosphate and calcium metabolism within human body (14). It stands as the foremost marker for the objective assessment of vitamin D µg status (15). Meta-analyses have already substantiated a correlation between low levels of 25(OH)D and an escalated risk of ischemic stroke (16, 17). Acute stroke patients often exhibit reduced 25(OH)D levels, which has been identified as an independent prognostic marker for mortality and poorer functional outcomes within 90 days after acute stroke (18). Furthermore, studies have identified a connection between migraine and serum vitamin D levels, with results indicating lower vitamin D concentrations in migraine patients than in healthy individuals (19–21). Research by Liampas et al. demonstrated an association between vitamin D and TTH, but the causal nature of this association and the efficacy of vitamin D supplementation in preventing TTH remain unclear (22). Therefore, investigating the relationship between 25(OH)D and neurological disorders such as migraine and severe headaches is of paramount importance for both headache prevention and treatment.

The differences in serum 25(OH)D levels between migraine patients and healthy individuals have been evidenced, with results showing significantly lower levels of 25(OH)D in the serum of migraine patients than in the control group (21, 23). Thus, the specific association between different concentrations of 25(OH)D and migraine or severe headaches remains to be explored. Therefore, based on the National Health and Nutrition Examination Survey (NHANES) database, our study hypothesized a negative correlation between serum 25(OH)D levels and severe headaches or migraines. Our study investigated for the first time whether serum 25(OH)D is indeed associated with severe headaches or migraines to validate our hypothesis. We also examined the levels of serum 25(OH)D that significantly impact severe headaches or migraines. Through this study, we hope to provide some data to support the evaluation of the beneficial effects of vitamin D supplementation on severe headaches or migraines.

## Methods

### Study population

National Health and Nutrition Examination Survey, a representative survey evaluating the health and nutritional status of the U.S. population, furnishes comprehensive data encompassing equitable demographic, dietary, laboratory, and questionnaire information. NHANES data collection involves household interviews, mobile examinations, and laboratory tests and is made accessible to data researchers and users. NHANES samples are selected using a multistage, stratified, probability sampling method, ensuring that each survey cycle’s sample is nationally representative (24). The survey participants for each cycle are randomly selected, with no follow-up tracking of participants. This study used samples from two cycles of the NHANES database: 2001–2002 and 2003–2004. The patients selected in these two cycles were different, and the data collected represented cross-sectional data for participants in each specific cycle. The NHANES survey protocol was approved by the National Center for Health Statistics Ethics Review Board (NCHS), and all participants provided informed consent. Details related to the methodology and data collection can be freely accessed on the NHANES website (http://www.cdc.gov/nchs/nhanes.htm).

**Fig. 1:**
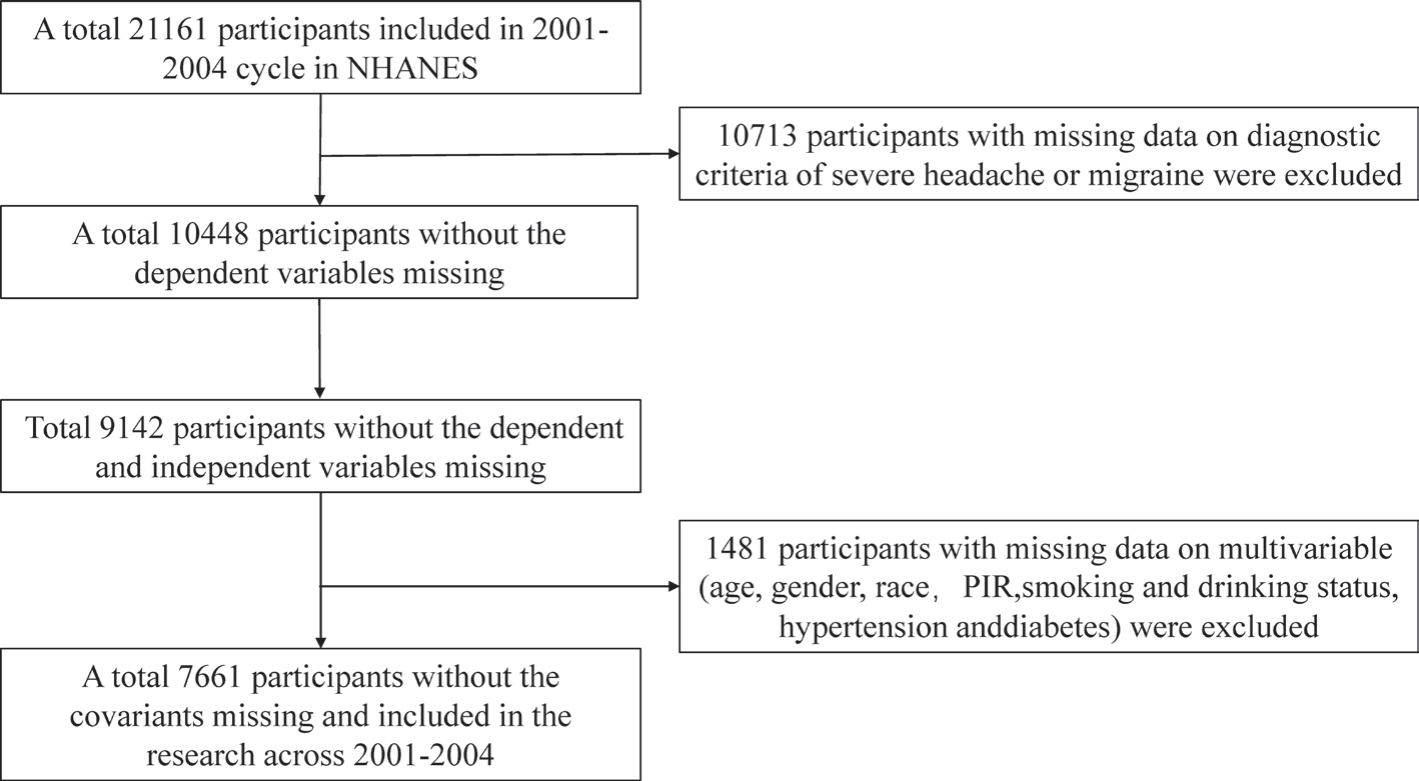
Sample selection flow chart for NHANES 2001–2004.

Initially, our investigation encompassed 21,161 participants, gathering available data pertaining to migraines, severe headaches, and serum 25(OH)D. After exclusion of participants lacking diagnostic criteria for severe headaches or migraines (*n* = 10,713), those with missing serum 25(OH)D concentration data (*n* = 1,306), as well as participants lacking data on age, gender, race, Poverty Index Ratio (PIR), smoking and alcohol status, hypertension, and diabetes (*n* = 1,481), a final total of 7,661 participants were contained in the statistical analysis for our study (Fig. 1).

### Serum 25(OH)D measurement

It was measured using DiaSorin RIA kit and converted to equivalent 25(OH)D concentrations through regression methodology, employing standardized liquid chromatography-tandem mass spectrometry for measurement (25).

### Headache identification

Severe headaches or migraines were defined by positive responses to the question, ‘Have you experienced severe headaches or migraines in the past 3 months?’ (26).

### Covariates

Standardized questionnaires were utilized to collect demographic characteristics of each respondent, encompassing age, gender, race, and poverty income ratio (RIAGENDR, RIDAGEYR, RIDRETH1, and INDFMPIR), along with information on smoking, alcohol consumption, and comorbidities. This study analyzed the population aged 20 years and older. The following racial and ethnic categories were employed: Mexican American, Other Hispanic, Non-Hispanic Black, Non-Hispanic White, and Other Race groups. The PIR was categorized as ≤1.3 (low income, where the family’s total income is less than or equal to 1.3 times the poverty line), 1.3–3.5 (moderate income, where the family’s total income falls between 1.3 and 3.5 times the poverty line), and >3.5 (high income, where the family’s total income exceeds 3.5 times the poverty line) (27). Alcohol consumption is defined as answering ‘Yes’ to the question ‘Had at least 12 alcohol drinks/1 yr?’ (where one drink refers to 12 ounces of beer, 5 ounces of wine, or 1.5 ounces of distilled spirits) (28). Smoking status was defined as follows (29): respondents who responded ‘Every day’ or ‘Some days’ to the question ‘Do you now smoke cigarettes?’ were classified as current smokers; those responding ‘Yes’ to ‘Smoked at least 100 cigarettes in life’ were classified as ever smokers; the remainder were categorized as never smokers. Hypertension (30) was defined by the following criteria: (1) previous diagnosis of hypertension; (2) in the NHANES examination section, average systolic blood pressure ≥130 mmHg or diastolic blood pressure ≥80 mmHg (Three consecutive blood pressure readings were obtained after the participants had sat quietly for 5 min, and the maximum inflation level had been determined. In cases where patients have undergone three measurements, the initial systolic and diastolic readings are excluded. The average of the second and third readings is then computed as the mean blood pressure. When only two readings are accessible, solely the second reading is designated as the mean blood pressure. In scenarios where only one reading is captured, it is utilized as the mean blood pressure value.) (30, 31). The diastolic blood pressure threshold was 80, and the systolic blood pressure threshold was 130. Diabetes was defined by the following criteria (32): 1) diagnosis of diabetes by a medical practitioner; 2) current usage of antidiabetic medication; 3) glycated hemoglobin >6.5%; 4) fasting blood glucose >126 mg/dL. Dietary calcium and fat intake data were collected through 24-h dietary recall surveys in the NHANES database. Participants underwent two dietary recalls: the first recorded at mobile examination centers and the second obtained through telephone follow-up 3–10 days later. Dietary calcium and fat intake for each participant were then calculated based on these dietary records using the Food and Nutrient Database for Dietary Studies provided by the United States Department of Agriculture. For datasets with no missing values in both recalls, the mean intake was calculated (https://wwwn.cdc.gov/Nchs/Nhanes/2003-2004/DR1TOT_C.htm#).

### Statistical analysis

In this study, R software (version 4.2) was utilized for all data analyses. The baseline table was generated using the ‘tableone’ package (https://cran.r-project.org/web/packages/tableone/index.html), stratified by whether participants suffered from severe headaches or migraines. Categorical variables were presented as sample size and proportion (n(%)), while continuous variables were presented as mean and standard deviation (mean[SD]), with adjustment for weighting in n(%), mean, and SD. A weighted logistic regression model was constructed using the ‘survey’ package to examine the association between serum 25(OH)D concentration and severe headaches or migraines. Stratified analysis of categorical variables was conducted in the model without adjusting for confounders. Further interaction analysis was performed by adjusting for all confounders, with chi-square tests used to determine statistical significance (*P* < 0.1 indicating significant differences). Serum 25(OH)D concentration was treated as a continuous variable and stratified by quartiles. Weighted logistic regression models were built using the ‘survey’ package to investigate the association between serum 25(OH)D and severe headaches or migraines, adjusting for various confounders. Crude adjustments were made in model I, while model II adjusted for gender, age, race, PIR, smoking, and alcohol consumption, and model III additionally adjusted for diabetes and hypertension.

Subsequent analyses explored the impact of serum 25(OH)D concentration on migraines or severe headaches in different populations, focusing on meaningful factors from interaction analysis (smoking and hypertension) and gender (33). Weighted logistic regression models were constructed using the ‘survey’ package, followed by subgroup analysis in different populations, with confounder adjustment as mentioned above. Furthermore, RCS was employed to investigate the relationship between serum 25(OH)D concentration and severe headaches or migraines in different populations. RCS is a commonly used method for fitting non-linear relationships between independent and dependent variables. It involves dividing the data into intervals and using a cubic polynomial to fit the data within each interval. Additional constraints are applied to ensure specific properties of the model within certain intervals, enhancing interpretability, stability, and preventing overfitting (34). Results were presented as odds ratios (OR) and 95% confidence intervals (CI), with *P* < 0.05 considered statistically significant.

## Results

A total of 7,661 respondents were ultimately included. Detailed baseline characteristics of all subjects here were listed in Table 1. The data revealed that 1,576 individuals (22.3%) suffered from migraines or severe headaches. Significant differences (*P* < 0.05) were observed between the migraine/severe headache and non-migraine/non-severe headache groups in terms of gender, age, race, PIR, smoking status, alcohol consumption, the presence of hypertension and diabetes, as well as serum 25(OH)D concentration. The majority of participants with migraines or severe headaches were in the age range of female, of middle to high income, and non-smokers (all *P* < 0.001). Furthermore, a notable portion of participants with migraines or severe headaches had a history of alcohol consumption or hypertension. Additionally, the serum 25(OH)D concentration was lower in the group experiencing migraines or severe headaches (61.00 vs. 63.55, *P* = 0.003) (Table 1).

**Table 1:** Baseline characteristics of all subjects in the 2001-2004 NHANES

Characters	Total	Non-headache	headache	*P* Value
**Overall**	7661 (100)	6085 (77.7)	1576 (22.3)	
**Gender**				< 0.001
Female	3930 (51.1)	2883 (47.0)	1047 (65.3)	
Male	3731 (48.9)	3202 (53.0)	529 (34.7)	
**Age**	45.72 (16.48)	46.98(16.94)	41.36 (13.91)	< 0.001
**Race**				0.229
Mexican American	1591 (7.4)	1253 (7.3)	338 (7.4)	
Other Hispanic	261 (4.3)	196 (4.0)	65 (5.3)	
Non-Hispanic White	4173 (74.0)	3367 (74.7)	806 (71.5)	
Non-Hispanic Black	1375 (10.0)	1061 (9.6)	314 (11.5)	
Other race	261 (4.4)	208 (4.4)	53 (4.2)	
**PIR**				< 0.001
≤1.3	2055 (19.6)	1512 (17.5)	543 (26.9)	
1.3-3.5	2976 (36.2)	2363 (35.5)	613 (38.8)	
> 3.5	2630 (44.2)	2210 (47.0)	420 (34.4)	
**Smoking**				< 0.001
Never smoking	3862 (49.6)	3061 (50.0)	801 (48.3)	
Former smoking	2084 (25.5)	1758 (27.0)	326 (20.2)	
Now Smoking	1715 (24.9)	1266 (23.1)	449 (31.5)	
**Alcohol drinking**				0.015
No	2353 (27.1)	1795 (26.1)	558 (30.5)	
Yes	5308 (72.9)	4290 (73.9)	1018 (69.5)	
**Hypertension**				0.01
No	3576 (50.9)	2747 (50.2)	829 (53.6)	
Yes	4085 (49.1)	3338 (49.8)	747 (46.4)	
**Diabetes**				0.144
No	6896 (93.0)	5458 (92.8)	1438 (93.7)	
Yes	765 (7.0)	627 (7.2)	138 (6.3)	
**25-Hydroxyvitamin D(nmol/L)**	62.98 (23.14)	63.55 (23.11)	61.00 (23.14)	0.003

Note: Categorical variables are presented as n(%) and continuous variables as mean (sd). N is unweighted; n(%), mean and sd are adjusted for weighted values.

Table 2 presents the findings from weighted logistic regression models investigating the association between serum 25(OH)D and severe headaches or migraines. Stratified analysis without adjusting for confounders provided clearer estimates of the effect of serum 25(OH)D on the risk of severe headaches or migraines in specific populations. The results indicated a decreased risk of migraines or severe headaches with increasing serum 25(OH)D concentration in subgroups such as the female population (OR: 0.995, 95% CI: 0.991–0.998, *P* = 0.001), never smokers (OR: 0.991, 95% CI: 0.985–0.997, *P* = 0.003), and non-drinkers (OR: 0.993, 95% CI: 0.987–0.999, *P* = 0.022).

**Table 2:** Association between serum 25-Hydroxyvitamin D and severe headache or migraine

Participants	OR	95% CI1	P-value	P for interaction
**Gender**				0.253
Female	0.995	0.991-0.998	0.001	
Male	0.998	0.991-1.004	0.483	
**PIR**				0.580
≤1.3	0.997	0.990-1.003	0.246	
1.3-3 5	0.997	0.990-1.003	0.244	
> 3.5	0.997	0.991-1.002	0.221	
**Race**				0.196
Mexican American	0.991	0.979-1.003	0.12	
Other Hispanic	0.996	0.983-1.010	0.571	
Non-Hispanic White	0.997	0.992-1.001	0.098	
Non-Hispanic Black	0.992	0.987-1.001	0.053	
Other race	0.989	0.971-1.008	0.24	
**Smoking**				0.003
Never smoking	0.991	0.985-0.997	0.003	
Former smoking	0.997	0.990-1.003	0.294	
Now Smoking	1.001	0.996-1.007	0.606	
**Alcohol drinking**				0.778
No	0.993	0.987-0.999	0.022	
Yes	0.997	0.993-1.001	0.08	
**Hypertension**				0.054
No	0.994	0.989-0.998	0.002	
Yes	0.996	0.992-1.000	0.07	
**Diabetes**				0.567
No	0.995	0.992-0.998	0.002	
Yes	0.989	0.979-1.000	0.044	

Note: P values for interaction terms were adjusted for gender, age, race, PIR, smoking, alcohol consumption, hypertension, and diabetes.

To ensure the accuracy and reliability of the effect estimates, further interaction analysis was conducted by adjusting for confounders. The results revealed statistically significant *P*-values in the interaction term of smoking and hypertension (*P* for interaction < 0.1) (Table 2).

We conducted separate analyses treating 25(OH)D as both a continuous variable and a categorical variable (stratified by quartiles) to explore the impact of serum 25(OH)D on the risk of severe headaches or migraines. The results for the continuous variable are shown in Table 3, indicating a significant negative correlation between serum 25(OH)D and the risk of severe headaches or migraines in all models (*P* < 0.05). This suggested that an increase in serum 25(OH)D significantly reduced the risk of experiencing severe headaches or migraines. Further analysis stratified by quartiles, with Q1 (≤46.8) as reference, showed that, except for Model III, all other models demonstrated a significant negative correlation between serum 25(OH)D concentration and the risk of severe headaches or migraines (*P* < 0.05). Additionally, as serum 25(OH)D concentration increased, the risk of severe headaches or migraines showed a significant decreasing trend. Furthermore, in subsequent analyses, we included other dietary factors (dietary calcium intake and dietary fat intake) to assess their impact on the relationship between serum 25(OH)D and severe headaches or migraines (Table 1). The results further confirmed a significant negative correlation between 25(OH)D and the risk of severe headaches or migraines (*P* = 0.046).

**Table 3:** Association of serum 25-hydroxyvitamin D concentrations with severe headache or migraine

Participants	OR (95% CI)			
	Crude	Model I	Model II	Model III
**25-Hydroxyvitamin D (continuous)**	0.995 (0.992-0.998)	0.995 (0.991-0.998)	0.996 (0.992-1.000)	0.996 (0.992-1.000)
*P-value*	0.002	0.003	0.047	0.049
**25-Hydroxyvitamin D (categorical)**				
Q1 (≤ 46.8, N = 2499)	Ref.	Ref.	Ref.	Ref.
Q2 (46.8-61.1, N =1911)	0.808 (0.679-0.960)	0.870 (0.736-1.030)	0.916 (0.770-1.089)	0.928 (0.781-1.102)
Q3 (61.1-75.4, N =1706)	0.769 (0.632-0.936)	0.821 (0.675-0.998)	0.863 (0.709-1.050)	0.883 (0.724-1.075)
Q4 (> 75.4, N = 1545)	0.742 (0.606-0.908)	0.740 (0.583-0.939)	0.782 (0.614-0.995)	0.809 (0.630-1.040)
*P* ***_trend_***	0.002	0.011	0.035	0.077

Note: Crude oil has not been adjusted; model I adjusted for gender, age, and race. model II adjusted for gender, age, race, PIR, smoking, and drinking; model III adjusted for gender, age, race, PIR, smoking, alcohol consumption, diabetes, and hypertension.

Considering the significant interaction results (*P* < 0.1) from stratified analysis, we further conducted subgroup analysis, focusing on factors such as smoking, hypertension, and gender [previously shown in relevant literature to have significant differences in migraine prevalence (33)]. The results (Table 4) indicated that, in Model III, a significant negative correlation was observed between serum 25(OH)D and the risk of severe headaches or migraines in subgroups including females (OR: 0.995, 95% CI: 0.991–0.999, *P* = 0.007), never smokers (OR: 0.993, 95% CI: 0.987–0.999, *P* = 0.018), and non-hypertensive individuals (OR: 0.994, 95% CI: 0.989–0.999, *P* = 0.018).

**Table 4:** Effect of 25-hydroxyvitamin D concentration on migraine or severe headache in different populations

25-Hydroxyvitamin D (OR (95%CI), *p* value)	Crude	Model I	Model II	Model III
**Gender**				
Female	0.995 (0.991-0.998), 0.001	0.994 (0.990-0.997), <0.001	0.995 (0.991-0.999), 0.007	0.995 (0.991-0.999), 0.007
Male	0.998 (0.991-1.004), 0.483	0.997 (0.990-1.004), 0.381	0.998 (0.990-1.006), 0.614	0.998 (0.990-1.006), 0.648
**Smoking**				
Never smoking	0.991 (0.985-0.997), 0.003	0.992 (0.985-0.998), 0.009	0.992 (0.986-0.999), 0.013	0.993 (0.987-0.999), 0.018
Former smoking	0.997 (0.990-1.003), 0.294	0.995 (0.987-1.002), 0.154	0.996 (0.989, 1.003), 0.223	0.996 (0.989, 1.004), 0.277
Now Smoking	1.001 (0.996-1.007), 0.606	1.000 (0.994-1.007), 0.957	1.000 (0.993-1.007), 0.940	1.001 (0.994-1.008), 0.837
**Hypertension**				
No	0.994 (0.989-0.998), 0.002	0.993 (0.988-0.998), 0.003	0.994 (0.989-0.999), 0.017	0.994 (0.989-0.999), 0.018
Yes	0.996 (0.992-1.000), 0.070	0.998 (0.993-1.003), 0.421	0.999 (0.994-1.004), 0.711	0.999 (0.994-1.005), 0751

Note: Crude oil has not been adjusted; model I adjusted for gender, age, and race. model II adjusted for gender, age, race, PIR, smoking, and drinking; model III adjusted for gender, age, race, PIR, smoking, alcohol consumption, diabetes, and hypertension

**Fig. 2:**
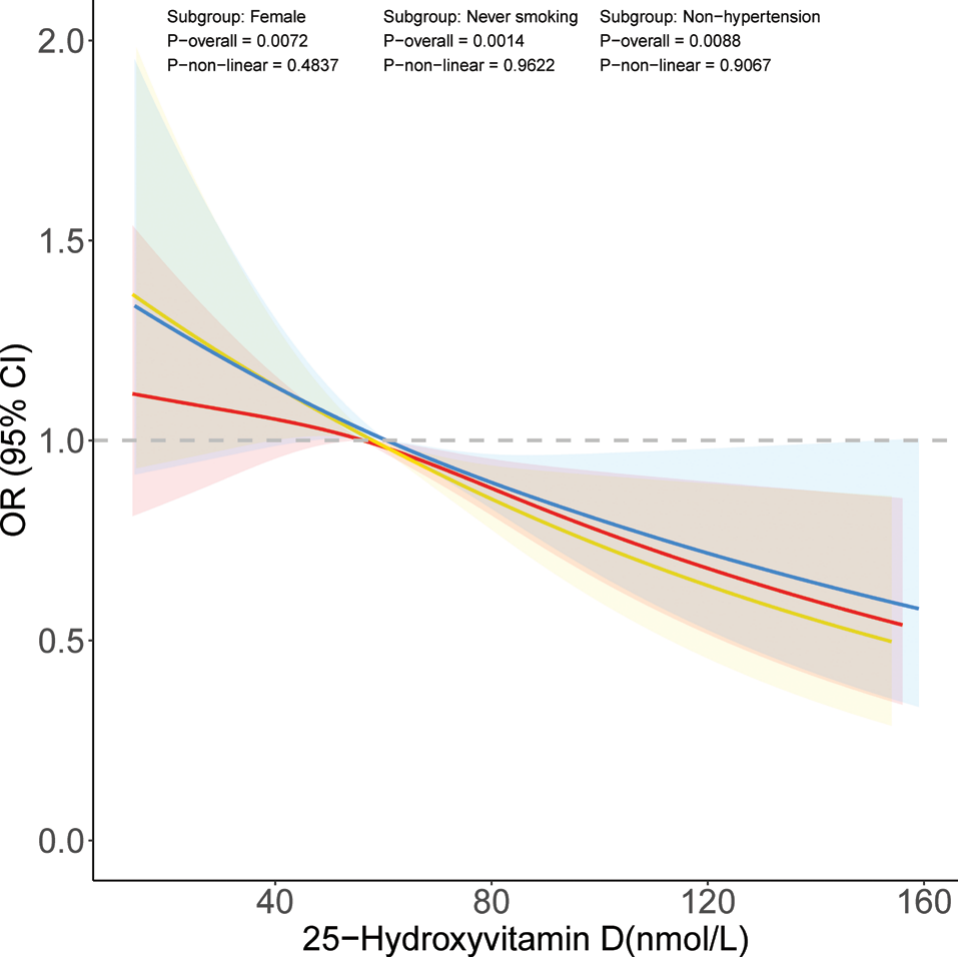
Restricted cubic spline analysis of the relationship between 25-hydroxyvitamin D concentration and the occurrence of severe headache or migraine in different populations. Female: red; never smoking: yellow; non-hypertension: blue.

Furthermore, based on the subgroup analysis, we conducted RCS analysis (Fig. 2) to explore whether there is a non-linear relationship between serum 25(OH)D concentration and the occurrence of severe headaches or migraines in the aforementioned significant subgroups. The results suggested that in females, never smokers, and non-hypertensive individuals, there may be a significant linear relationship between serum 25(OH)D concentration and the presence of severe headaches or migraines (*P*-non-linear > 0.05 and *P*-overall < 0.05).

## Discussion

Our study was a research endeavor ground on the NHANES database to mine the linkage between 25(OH)D and the risk of migraines or severe headaches. Our study findings indicated a negative correlation between serum 25(OH)D levels and the occurrence of migraines or severe headaches. Moreover, this association appears to be more pronounced in females, never smokers, and individuals without hypertension.

Migraines are complex disorders that significantly impact daily life. Due to limited efficacy of current pharmacological and non-pharmacological treatments, the prevention and management of migraines remain challenging (35). Mounting empirical indications posit an intimate correlation between insufficiency in 25(OH)D and the manifestation of migraines. Some studies have found a higher number of headache days per month to be associated with vitamin D µg deficiency among migraine sufferers (36). Additionally, aerobic training combined with vitamin D µg supplementation has been reported to produce synergistic effects, providing extra psychological and cognitive health benefits for males experiencing migraines and vitamin D µg deficiency (37). Supplementation with vitamin D µg at doses of 1,000–4,000 IU/day can also decrease the frequency of migraine attacks (19). Furthermore, observational study findings suggested a connection between 25(OH)D deficiency and chronic headaches. The underlying mechanism could be attributed to the role of inflammation, which is commonly considered one of the fundamental triggers of migraines. Inflammation activates the trigeminal nerve, a major structure implicated in migraines. Vitamin D µg can contribute to alleviating pain in migraine sufferers. Diminution in the secretion of pro-inflammatory cytokines and inhibition of T-cell responses have been identified as key mechanisms through which vitamin D µg enacts its regulatory effects.

Recent studies have also revealed an association between migraine indices and serum vitamin D µg concentrations, indicating lower vitamin D µg levels in migraine sufferers compared to healthy individuals. Conversely, elevated vitamin D µg concentrations appear to confer a protective effect for migraine patients (19–21). The present study similarly demonstrated a negative correlation between vitamin D µg and the occurrence of migraines or severe headaches, with the risk of these conditions decreasing as serum 25(OH)D levels rise. These findings aligned with prior related research, suggesting that vitamin D µg might exert regulatory effects within the brain in primary headaches like migraines through various mechanisms. These mechanisms encompass gene downregulation linked to cell apoptosis, resulting in increased cell growth, modulation of different neurotrophic factors like nerve growth factor for neuroprotective effects, acting as a potent antioxidant supporting cerebral vascular health, and influencing several brain neurotransmitters, including dopamine, acetylcholine, and serotonin, to alleviate migraines (38).

Prior relevant studies have indicated that gender is a crucial factor influencing primary headaches and 25(OH)D levels. Research on the role of vitamin D deficiency in childhood primary headaches suggests that compared to healthy children without headaches, girls with primary headaches have significantly lower levels of 25(OH)D than boys, suggesting that female gender may be considered a negative factor for primary headaches (39). The findings of our study revealed a more pronounced association between 25(OH)D and the occurrence of migraines or severe headaches in females, which aligns with the aforementioned research. This could be attributed to the fact that within the same age group, females inherently have a higher prevalence of migraines or severe headaches than males (33, 40). Despite evident epidemiological differences in migraines between genders, the current understanding of the underlying mechanisms of this pattern remains limited. However, sex hormones are believed to be a significant factor influencing the pathophysiology of migraines and the natural course of migraines throughout life (41). Hence, we speculated that the gender differences contributing to the association between 25(OH)D and the occurrence of migraines or severe headaches may be related to potential estrogen effects.

Moreover, recent studies have suggested a complex and multifactorial connection between migraines and hypertension, with more conclusions leaning toward migraines being a potential risk factor for hypertension. For instance, a cohort study indicated that compared to females without migraines, females with migraines have a higher relative risk of developing hypertension (42). There have also been discussions regarding the link between 25(OH)D and hypertension, with low concentrations of 25(OH)D found to be associated with elevated blood pressure (43, 44). Vitamin D supplementation significantly increases 25(OH)D concentrations, seemingly aiding in blood pressure reduction, particularly in elderly individuals with elevated blood pressure and vitamin D deficiency (45, 46). This may be related to the activation of the renin-angiotensin system, abnormal nitric oxide regulation, oxidative stress, or alterations in inflammatory pathways following vitamin D deficiency (47). However, a population-based cross-sectional study found no association between low concentrations of 25(OH)D and hypertension (48). Currently, it remains unclear whether the relationship between low concentrations of 25(OH)D and increased risk of hypertension is causal, necessitating further research for confirmation. Based on these studies, it can be inferred that among non-hypertensive individuals, migraine patients with severe symptoms may alleviate migraine symptoms by directly or indirectly increasing 25(OH)D concentrations.

Sustained healthy diet, aerobic exercise, and continuous low stress are common lifestyle recommendations for migraines (49–52). It is well-known that smoking, as an unhealthy lifestyle, has widespread negative impacts on health. However, research on the relationship between smoking and migraine attacks is relatively scarce, and the results are inconsistent. For example, a large-scale study (980 individuals) conducted in New Zealand found that smoking during adolescence does not increase the risk of headaches in adulthood (53). Conversely, a recent Mendelian randomization study found that smoking increases the risk of migraines (54). The results of our study support the association between smoking and increased risk of migraines, but the role of smoking in the relationship between 25(OH)D and the occurrence of migraines or severe headaches requires further investigation.

Related research indicates that dietary calcium intake has a certain impact on the response of serum 25(OH)D after vitamin D supplementation. Studies have shown that combined supplementation of 1,000 IU of vitamin D + 1,000 mg of calcium is more effective in increasing serum 25(OH)D concentrations than using vitamin D or calcium alone (55). Furthermore, a related study on postmenopausal African American women with vitamin D deficiency supplementing with vitamin D showed that for every 1,000 mg increase in calcium intake, serum 25(OH)D concentration increased by 3.8 ng/mL (56). The related mechanism may be that calcium supplementation inhibits the metabolism of 25OHD, primarily by altering its half-life to increase serum 25OHD levels (55). Vitamin D is a fat-soluble vitamin, and a moderate intake of fat in the diet may aid its absorption (57). Studies have reported a positive correlation between the content of monounsaturated fatty acids and the increase in serum 25(OH)D after vitamin D supplementation (58). Therefore, healthy dietary habits and lifestyle are crucial for influencing the body’s vitamin D levels, which also explains how increasing calcium and fat intake in the diet can raise serum 25(OH)D concentrations, thereby playing a certain role in preventing severe headaches or migraines.

Our study explores concentrations of 25(OH)D associated with reduced risk of severe headaches or migraines, which are equal to or greater than 46.8 nmol/L. Studies have indicated that achieving a concentration of 62.4 nmol/L of 25(OH)D can lower the risk of chronic headaches (20). Beyond migraines, research on neonatal neurocognitive development suggests that the optimal level of 25(OH)D should be between 30 and 50 nmol/L (59). Considering the importance of vitamin D in regulating the activation, proliferation, and differentiation of inflammation processes in migraines or other neurological diseases, the significant variation in beneficial concentrations of 25(OH)D could be due to the heterogeneity of different populations. Moreover, according to the Endocrine Society, the optimal concentration of serum 25(OH)D in the general adult population should be at least 75.00 nmol/L to achieve better health outcomes (60). This suggests that reaching a certain level of serum 25(OH)D has protective implications for specific populations, while also indicating varying needs for vitamin D among different groups. Although there is still controversy regarding the optimal concentration levels of serum 25(OH)D, it is recommended to increase vitamin D intake and have reasonable exposure to sunlight. Based on our study’s results, maintaining serum 25(OH)D at least at 46.8 nmol/L, preferably above 75.4 nmol/L, is suggested to achieve optimal overall health benefits of vitamin D. Additionally, we believe that there should be reasonable national and international programs to educate the public about the health benefits of vitamin D, formulate corresponding policies, conduct further research, fortify commonly consumed foods with vitamin D, and, thus, reduce the risk of neurological disorders such as migraines or severe headaches.

In summary, this work collected data from NHANES and nutrition examination survey, resulting in more comprehensive and reliable outcomes. Furthermore, the subgroup analysis unmasked a more significant association between migraines or severe headaches and 25(OH)D specifically in non-hypertensive individuals, a finding not previously reported in existing literature. However, limitations existed. First, given the cross-sectional design of this work, we are precluded from deducing a causal linkage between vitamin D µg deficiency and migraines from the extant dataset. Future research should explore this direction, possibly through prospective cohort studies. Second, despite our efforts to search the literature and adjust for potential confounding factors, given the complex, multifactorial nature of migraines, there remains the possibility of yet unrecognized or unquantified confounding factors in the etiology of migraines. As such, our current study might remain incomplete. Finally, the NHANES database does not explicitly record migraine subtypes and severity, preventing differentiation among types of headaches.

## Data Availability

This study used the National Health and Nutrition Examination Survey (NHANES) (http://www.cdc.gov/nchs/nhanes.htm) to collect data.
